# Affective Instability and Regular Cannabis Use During Adolescence and Subsequent Risk for Psychosis in Early Adulthood: A Longitudinal Birth Cohort Study

**DOI:** 10.1093/schizbullopen/sgag008

**Published:** 2026-03-18

**Authors:** Mrunal Bandawar, Matthew J Taylor, Isabel Morales-Muñoz, Steven Marwaha, Rachel Upthegrove

**Affiliations:** Institute for Mental Health, School of Psychology, University of Birmingham, West Midland B15 2TT, Birmingham, United Kingdom; Atlantic Recovery Centre, Change Grow Live, West Midland DY9 8EL, Dudley, United Kingdom; Mental Health Research Unit, Sheffield Centre for Health and Related Research, The University of Sheffield, South Yorkshire S14DA, United Kingdom; Sheffield Health & Social Care NHS Foundation Trust, Sheffield, South Yorkshire, S91US, United Kingdom; Institute for Mental Health, School of Psychology, University of Birmingham, West Midland B15 2TT, Birmingham, United Kingdom; Institute for Mental Health, School of Psychology, University of Birmingham, West Midland B15 2TT, Birmingham, United Kingdom; Specialist Mood Disorders Clinic, Birmingham and Solihull Mental Health NHS Trust, Birmingham, West Midland, B138QY, United Kingdom; Department of Psychiatry, University of Oxford, Oxford, Oxfordshire, OX37JX, United Kingdom; Early Intervention Service, Forward Thinking Birmingham, Birmingham and Solihull Mental Health NHS Trust, Birmingham, West Midland, B191HS, United Kingdom

**Keywords:** ALSPAC, schizophrenia, marijuana, emotion

## Abstract

**Background and Hypothesis:**

Affective instability (AffI) is associated with psychosis, and cannabis use is a known risk factor for psychosis. Previous evidence suggests that AffI may itself be a risk factor for cannabis use. This study aimed to investigate the longitudinal relationship between AffI and cannabis use during adolescence and the development of psychosis in early adulthood.

**Study Design:**

Data from the Avon Longitudinal Study of Parents and Children were analyzed to estimate the prevalence of AffI at age 12 and cannabis use frequency at ages 13, 14, 15, 17, 20, and 22. Psychotic experiences (PEs) and psychotic disorder (PD) were measured at age 24 using the Psychosis-like Symptoms Interview. Latent class growth analysis identified cannabis use trajectories and odds ratios (OR) were calculated to assess associations between adolescent AffI, different cannabis use patterns and psychotic outcomes. Path analysis was used to examine cannabis use as a mediator between AffI and psychotic outcomes.

**Study Results:**

Individuals with AffI were significantly more likely to develop psychotic outcomes at age 24 [PEs: OR = 4.08 (2.35–7.08); PD: OR = 4.74 (2.00–11.27)]. Regular cannabis users from ages 13–22 had an increased likelihood of developing psychotic outcomes compared with non-users [PEs: OR = 4.40 (2.79–6.92); PD: OR = 3.13 (1.25–7.88)]. Regular cannabis use partially mediated the relationship between AffI and both PEs and PD.

**Conclusions:**

Adolescents with AffI are at a higher risk of developing psychosis in early adulthood. Regular cannabis use mediates this risk, suggesting the potential benefit of targeted prevention strategies for at-risk youth.

## Introduction

Affective instability (AffI) is a transdiagnostic symptom characterized by rapid and intense fluctuations in affect and difficult-to-regulate fluctuations in emotional state.^1^ Contemporary models conceptualize AffI as a core deficit in the dynamic processes underlying mood regulation, including heightened emotional reactivity, slower return to baseline, and impaired modulation of affective responses.^2–4^ These features are thought to disrupt adaptive mood regulation and increase vulnerability to a range of psychiatric symptoms during adolescence, with higher levels of AffI being noted in individuals with various psychiatric conditions, including psychotic disorders,^5^ depression,^6^ bipolar disorder,^7^ borderline personality disorder,^7^^,^^8^ and substance misuse.^9^ AffI has emerged as a potential risk factor for regular cannabis use, with preliminary evidence suggesting that associations between AffI and cannabis use may begin to develop during adolescence.^10^^,^^11^

Cannabis use has long been recognized as a significant risk factor in the development and exacerbation of psychosis, with studies indicating a two-to-four-fold increase in people with first episodes of psychosis (FEP) among regular cannabis users compared with non-users,^12^^,^^13^ with a potentially causal relationship.^14^ Multiple motivations may drive regular cannabis use, ranging from recreational consumption to self-medication.^15^ Individuals may turn to cannabis use as a coping mechanism to seek relief from nascent symptoms associated with psychosis,^16^ and the neurobiological underpinnings of this phenomenon suggest a link between cannabis use and the brain’s reward and motivation systems.^17^ Reciprocally, cannabis use has been implicated in some of the neurobiological changes thought to play a role in the development of psychosis, such as alterations in glutamatergic and dopaminergic neurotransmitter systems.^18–20^

A novel perspective on the interplay between AffI, cannabis use, and psychosis is that cannabis use may at least partially mediate the effect of AffI on the development of psychosis. For instance, it is possible that AffI may drive the adoption of cannabis use as an example of self-medication or as a coping mechanism, with its prolonged use further increasing the risk of psychosis.^21–24^ Investigating this mediation effect holds promise for elucidating the mechanisms underlying the progression to psychosis observed among adolescent cannabis users with AffI and informing target interventions in this population. One approach for advancing the current evidence base includes the utilization of large-scale data with longitudinal interrogation.

Adolescence is a developmental period marked by heightened emotional reactivity, increased reward sensitivity, and rapid neurodevelopmental change. These features may amplify the impact of both AffI and cannabis exposure on later mental health. This study had multiple aims. First, it aimed to evaluate the prospective association of AffI in early adolescence with the development of psychotic outcomes in early adulthood. Second, it aimed to classify patterns of cannabis use emerging during adolescence. Finally, it aimed to examine whether regular cannabis use throughout adolescence mediated the prospective associations between AffI and psychotic outcomes in early adulthood. We hypothesized that AffI is a risk factor for regular cannabis use during adolescence and that adolescent AffI is a risk factor for psychosis in young adulthood, with regular cannabis use mediating this association.

## Methods

### Participants

This study utilized data from the Avon Longitudinal Study of Parents and Children (ALSPAC) cohort, a prospective UK birth cohort study focusing on the determinants of development, health, and disease from childhood onwards.^25–27^ Pregnant women residing in Avon, UK, with expected delivery dates between April 1, 1991 and December 31, 1992 were invited to participate. Initially, 14 541 eligible pregnancies were enrolled, resulting in 14 062 live births and 13 988 children who were alive at 1 year of age. Efforts to enhance the sample were made when the oldest children reached ~7 years of age, leading to data availability for an additional 913 children for measures collected after age seven. The total sample size for analyses using any data collected after the age of seven is therefore 15 447 pregnancies, resulting in 15 658 fetuses. Of these 14 901 children were alive at 1 year of age. Study data were collected and managed using REDCap electronic data capture tools hosted at the University of Bristol, UK. REDCap (Research Electronic Data Capture) is a secure, web-based software platform designed to support data capture for research studies.^28^ Further information regarding the ALPSAC cohort, including all of the data that is available through a fully searchable data dictionary and a variable search tool, is available on the study website (https://www.bristol.ac.uk/alspac/researchers/our-data).

Ethical approval for the study was obtained from the ALSPAC Ethics and Law Committee and the Local Research Ethics Committees. Informed consent for the use of data collected via questionnaires and clinics was obtained from participants following the recommendations of the ALSPAC Ethics and Law Committee at the time.

### Measures

#### Affective Instability at 12 Years Old

AffI at 12 years was assessed using the Childhood Interview for DSM-IV Borderline Personality Disorder (CI-BPD).^29^ The questionnaire asked “do you have a lot of sudden mood changes?”, with the timescale for this symptom being “suffered this over the last several years” and accepted responses as a binary “Yes” or “No.” This measure has been used in several other studies to estimate AffI in various disorders^5^^,^^30^ and has face validity as an indicator of AffI. However, the item has not undergone formal psychometric validation as a standalone measure. Therefore, the measure should be interpreted as a brief indicator of sudden affective fluctuation rather than a comprehensive assessment of AffI. In these, AffI was defined as intense, brief episodes of sadness, anxiety, or irritability occurring at least 25% of the time. Interviewers categorized AffI as “Definitely present,” “Suspected,” or “Not present.” In this study, we created a binary variable of AffI, in which 1 represented “Definitely present” and 0 represented “Suspected” or “Not present.”

#### Cannabis Use from 13 to 22 Years Old

Cannabis use data were collected during ALSPAC Teen Focus clinic visits at ages 13, 15, and 17, with additional time points for information on cannabis use arising through self-reported questionnaire data at ages 14, 20, and 22. Respondents were assured responses were confidential, and Teen Focus clinic materials indicate some assessment components required parents or caregivers not to be present. Responses to one or more questions regarding cannabis use at each time point were categorized into no use, occasional use (less than once per week), and frequent use (once per week or more) at each time point, similar to a previous study.^31^ Details of the binary responses used in categorization are included in Supplementary Table S1.

#### Psychotic Outcomes at 24 Years Old

Psychotic experiences (PEs) occurring in the previous six months were assessed using the Psychosis-Like Symptom Interview (PLIKSi) at the age 24 clinic visit.^32^ The PLIKSi incorporates elements of the K-SADS and DISC-IV and its use in the ALSPAC cohort has been described in detail elsewhere.^33^ Briefly, it includes 12 core questions addressing key positive symptoms of psychosis (hallucinations, delusions, and thought interference). The PLIKSi was delivered in a semi-structured manner during which participants were initially asked whether, since age 12, they had ever experienced the symptom in question, with the interviewer subsequently cross-questioning to enable further categorization of potential psychotic experiences as either definitely present, suspected, or not present. The outcome timepoint of age 24 was selected to provide a clear distinction of 11 years between it and the independent measure of AffI at age 12, with consistent cannabis use established in this period. Similar to AffI, cases of PEs were defined as individuals with definite PE, whereas suspected and not present were classed as negative.

Psychotic disorders (PD) at 24 years were derived similar to previous research.^34^ PD was defined as being present when the participant experienced definite PEs not attributable to sleep or fever, which had recurred regularly over the previous 6 months, and had subjectively reported their symptoms as very distressing or having a very negative impact on their social or occupational functioning.

### Confounding Variables

Family risk factors were captured using the Family Adversity Index (FAI) at three intervals: during pregnancy (extended index), at 2 years postnatally (extended index), and at 4 years postnatally (short index). The FAI assesses factors such as early parenthood, housing and family conditions, maternal education, financial hardships, parental relationship, maternal mental health issues, substance abuse by parents, partner support, and social networks. Points were tallied at each interval to calculate a total FAI score. Total FAI score was included as a confounding variable, along with smoking at age 13, sex, gestational age, child ethnicity, and maternal age at childbirth. These variables were controlled due to their known associations with mental health outcomes.^30^^,^^31^^,^^34–36^

### Statistical Analysis

All statistical analyses were performed using SPSS version 28 (SPSS Inc., Chicago, IL, USA). Initially, a descriptive analysis was carried out for all relevant variables. In the main analysis, first, a latent class growth analysis (LCGA) was performed using MPlus v8 (Muthén&Muthén) to identify cannabis use trajectories from ages 13 to 22 (13, 14, 15, 17, 20, and 22 years), with the best-fitting classification model as determined using three indices (Bayesian information criteria [BIC], Vuong-Lo–Mendell–Rubin [VLMR] test, and entropy) being selected.^31^ The full information maximum likelihood (FIML) estimation method was used to manage missing values owing to attrition. Following this, binomial logistic regressions were conducted using SPSS to examine associations between AffI at 12 years and psychosis at 24 years, as well as associations between cannabis use trajectories and psychosis at 24 years. Following unadjusted associations, adjusted associations were tested controlling for the confounding variables. Model 1 (unadjusted) comprised logistic regression with AffI predicting PE or PD.

Model 2 (adjusted) comprised logistic regression including AffI, with sex, ethnic group, gestational age, maternal age, FAI, and cannabis use at age 13 as covariates. Odds ratios for the outcome measures among regular and occasional cannabis users are reported as compared with the reference group of cannabis non-users.

Missingness in the data was not at random. Missing data at age 24 favored males with younger maternal age at delivery, shorter gestational age, lower birth weight, and higher socioeconomic levels (Supplementary Table S2). Inverse probability weighting (IPW) was utilized to address the missingness. A logistic regression model was used to determine weights for each individual using the inverse probability of response depending on the variables associated with selective dropout. We utilized the regression coefficients derived from this model to calculate probability weights applied to the covariates in the primary analyses.

IPW was chosen because the probability of remaining in the cohort at age 24 was associated with baseline sociodemographic and perinatal variables, meaning data were not missing completely at random. IPW uses the inverse of the predicted probability of response to reweight participants and reduce attrition bias. FIML assumes missing at random conditional on observed data and is widely used for cohort studies with longitudinal repeated measures.

Path analysis was conducted using SPSS AMOS to explore the potential mediating role of cannabis use trajectories in the association between AffI at 12 and psychosis outcomes at age 24. Separate path analyses were conducted for each psychosis outcome (PEs and PD). The independent (presence vs absence of AffI), mediating (regular vs non-user of cannabis), and outcome (presence vs absence) variables were treated as dichotomous. The occasional cannabis users were not included in the comparison in this part of the initial analysis. Bootstrapped bias-corrected 95% confidence intervals and *P*-values were derived for assessing the significance of the standardized indirect associations. All analyses in AMOS were adjusted for the significant confounding variables from the regression models and missing data were managed using the FIML method.

In a sensitivity analysis, we treated cannabis trajectories as a three-level mediator (non-user [reference], occasional, regular). We estimated the mediator model using a baseline-category multinomial logit and the outcome model using logistic regression with indicator variables for occasional and regular use. Natural direct and indirect effects were obtained by g-computation, averaging over the empirical covariate distribution and bootstrap, bias-corrected 95% CI were computed. This analysis complements the primary regular-vs-non-user mediation by quantifying any pathway through occasional use.

## Results

This section first presents descriptive statistics and cannabis trajectory derivation, followed by analyses of the association between AffI and psychosis, the association between cannabis trajectories and psychotic outcomes, and mediation analyses. Of 14 901 children in ALSPAC, the available sample included 6404 with AffI at age 12, 6643 with cannabis data at age 13, and 3889 with psychosis outcome data at age 24. The number of cases with information available on cannabis use varied across time points between the ages of 13 and 22 years (Table 1). A total of 5704 (36.5%) participants had cannabis use assessed at three or more time points. The descriptive statistics of covariates and independent variables compared between groups of cannabis use are available in Supplementary Table S3.

**Table 1:** Prevalence of Cannabis Use at Each Time Point Estimated Using Available Data

Cannabis use	13 years (n = <6650^a^)	14 years (n = 6008)	15 years (n = 5307)	17 years (n = 3636)	20 years (n = 4189)	22 years (n = 3948)
Never	6560 (98.8%)	5798 (96.6%)	4791 (90.3%)	2449 (67.4%)	2032 (48.5%)	1884 (47.7%)
Occasional	77 (1.2%)	187 (3.1%)	356 (6.7%)	1014 (27.9%)	1919 (45.9%)	1871 (47.4%)
Frequent	<5^a^	20 (0.3%)	159 (3.0%)	172 (4.7%)	234 (5.6%)	192 (4.9%)
*Total*	6639	6005	5306	3635	4185	3947

a Number has been suppressed.

The available data for cannabis use ranged from 6643 participants at the age of 13 years to 3948 at the age of 22 years. Cannabis data for 5704 (36.5%) was available for three or more time points. The prevalence of both occasional and frequent cannabis use increased from age 13 to 22 years. The missing data pattern showed higher losses to follow-up with a higher family adversity index.

### Trajectories of Cannabis Use from 13 to 22 Years Old

**Figure 1:**
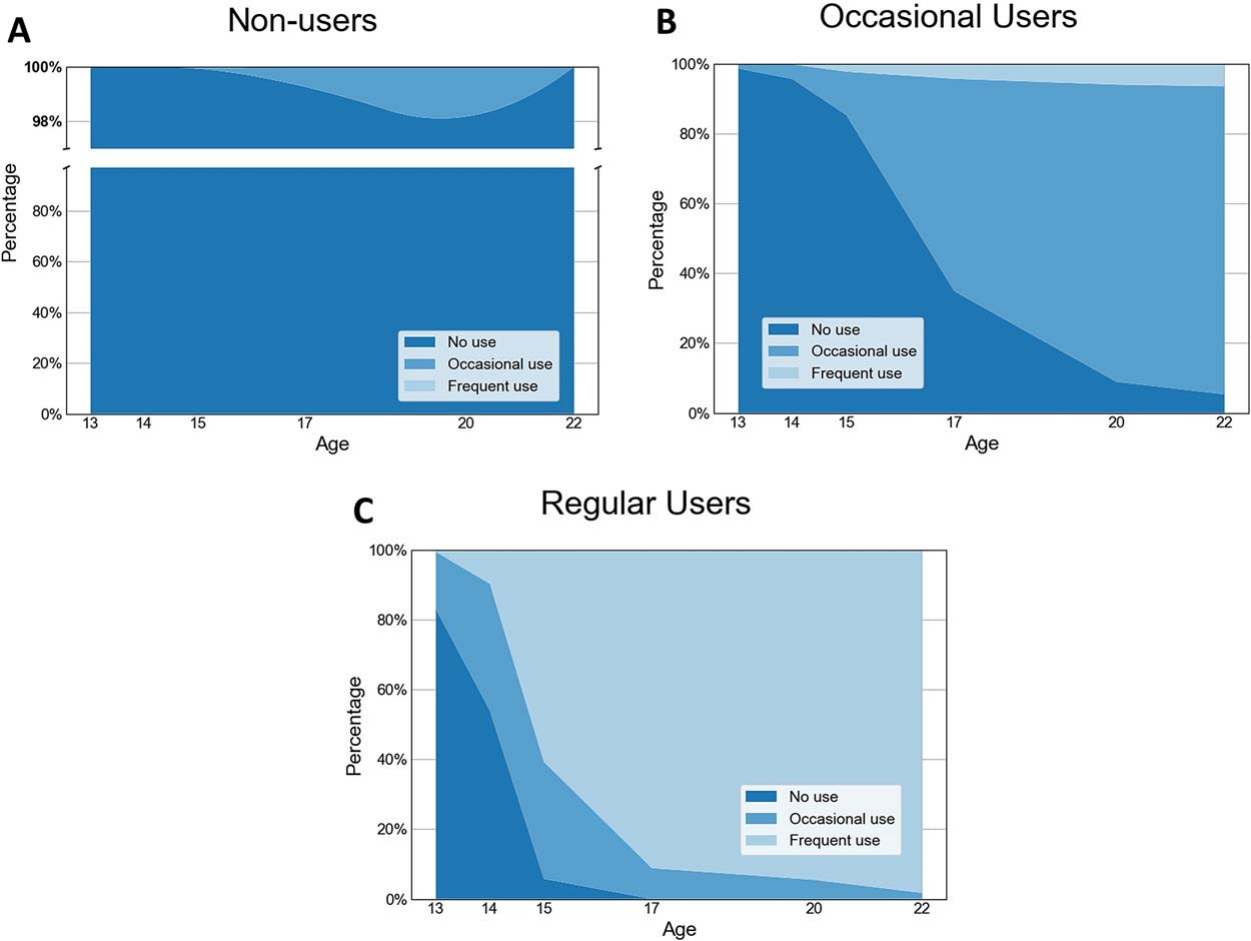
Profiles of Cannabis Use from the Latent Class Growth Analysis. Stacked Area Plots Showing the Change in Frequency of Cannabis Use Over Time Across Individuals Belonging Either to Non-Users (n = 2936) (A), Occasional Users (n = 2596) (B), or Regular Users (n = 171) (C). Y axis Interrupted in Figure 2A to Aid Visualization of Small Proportion of Respondents Indicating Occasional Cannabis Use.

Model fit indices with respect to VLMR, BIC, and entropy for all models assessed (2–6 classes) are presented in Supplementary Table S4. BIC decreased with the addition of each class indicating a better model fit with greater numbers of classes, a pattern typical of large samples.^37^ VLMR showed a statistically significant difference for the 2-class, 3-class, 4-class, and 6-class models, while VLMR was not statistically significant for 5-class. The entropy values for the 3-class model were the highest, compared with 2 or 6-class models, indicating the highest classification accuracy for the 3-class model. Taking this into account, the three main fit indices tested suggested that for cannabis use, the 3-class model offered the best overall model fit. To confirm that the 3-class solution was optimal for our data, we confirmed that group sizes contained >2% of the sample in each of the classes and that the trajectories detected were clinically relevant by corroborating this with the clinical experts in our study (SM and RU). The patterns of cannabis use identified in the 3-class model were subsequently labeled “Non-users” (n = 2936, 51.5%), “Occasional users” (n = 2596, 45.5%) and “Regular users” (n = 171, 3%). Figure 1 displays the proportion of respondents in each class who indicated no cannabis use, occasional cannabis use, or frequent cannabis use at each timepoint.

### Prevalence of AffI at 12 Years of Age and Associations Between AffI at 12 Years and Psychotic Outcomes at 24 Years

Of the 6094 children for whom data on AffI at age 12 were available, 310 (4.8%) reported definite AffI (Table 2). The weighted adjusted logistic regression model showed significantly higher odds ratios for both PEs (OR = 3.95 [95% CI 2.76–5.64], *P*<.001) and PD (OR = 2.49 [95% CI = 1.25 – 4.99], *P*<.05) at age 24 in individuals with definite AffI at 12. Among participants with AffI data at 12 years, the prevalence of definite AffI was 4.68% (121/2588) in those retained at the age 24 assessment and 4.8% (139/2906) among those lost to follow-up.

**Table 2:** Association Between Affective Instability at 12 Years and Psychotic Outcomes at 24 Years

Outcome at 24 years	Prevalence in whole sample (n/total)	Prevalence in AffI group (n/total)	Unadjusted OR (95% CI)	Adjusted OR (95% CI)
Psychotic experiences (PEs)	2.8% (91/3210)	13.6% (11/81)	4.03 (2.83–5.74)	3.95 (2.76–5.64)^***^
Psychotic disorder (PD)	1.2% (47/3210)	14.8% (<5/>20)	2.67 (1.34–5.31)	2.49 (1.25–4.99)^*^

AffI = affective instability (CI-BPD item); Percentages represent n/total participants with non-missing data for each outcome; PD cell counts in the AffI group are suppressed in accordance with ALSPAC disclosure control policy; Adjusted models include gender, ethnic group, family adversity index, maternal age at delivery, and gestational age at birth. Significance: *P*<.05 (^*^), *P*<.001 (^***^).

### Psychotic Outcomes at 24 Years in the Three Derived Trajectories of Cannabis Use

Overall, at age 24 years, 91 individuals (2.8%) reported psychotic experiences and 47 individuals (1.2%) developed psychotic disorder (Table 3). Compared with cannabis non-users during adolescence, occasional users were significantly more likely both to experience PEs (adjusted OR 1.34 [95% CI 1.03–1.74], *P*<.05) and to meet the criteria for PD (adjusted OR 1.96 [95% CI 1.25–3.07], *P*<.001) at 24 years of age. Regular cannabis users in adolescence displayed an even greater likelihood both of experiencing PEs (adjusted OR 4.40 [95% CI 2.79–6.92], *P*<.001) and meeting the criteria for PD (adjusted OR 3.13 [95% CI 1.25–7.88], *P*<.001).

**Table 3:** Psychotic Outcomes at 24 Years by Cannabis Use Trajectory

Cannabis trajectory group	Psychotic experiences (PEs) (% and n/total)	Adjusted OR for PEs (95% CI)	Psychotic disorder (PD) (% and n/total)	Adjusted OR for PD (95% CI)
Non-users (reference)	2.3% (35/1476)	—	0.9% (13/1498)	—
Occasional users	2.9% (46/1581)	1.34 (1.03–1.74)^*^	1.1% (18/1609)	1.96 (1.25–3.07)^**^
Regular users	13.9% (10/72)	4.40 (2.79–6.92)^***^	<5% (<5/69)	3.13 (1.25–7.88)^*^

Cannabis trajectory groups were derived using latent class growth analysis (ages 13–22); Percentages represent n/total participants with non-missing data for each outcome; PD case numbers for regular users are suppressed due to ALSPAC disclosure control policy; Adjusted odds ratios control for gender, ethnic group, family adversity index, maternal age at delivery, and gestational age at birth. Significance: *P*<.05 (^*^), *P*<.01 (^**^), *P*<.001 (^***^).

### Mediation Effect of Regular Cannabis Use in the Association Between AffI at 12 Years and Psychotic Outcomes at 24 Years

**Figure 2:**
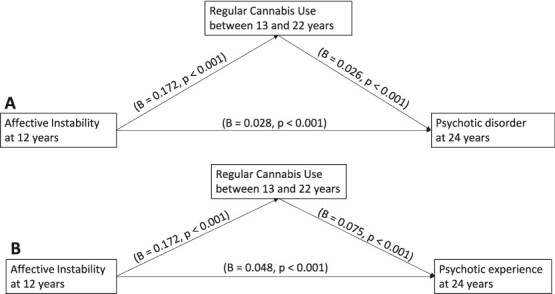
Path Diagram Displaying Main Direct Associations. Only the Direct Associations of the Independent Variable, Mediating Factor, and Dependent Variable are Shown. Affective Instability Represents the Independent Variable; Regular Cannabis Use Between Age 13 and 22 Represents the Mediating Factor; and Psychotic Disorder (A) and Psychotic Experiences (B) at Age 24 Represent the Outcomes. The Covariates Also Included in this Path Analysis Were Sex, Family Adversity, Birth Weight, and Smoking at Age 13 Years. Significant Pathways are Indicated by Solid Arrows.

In examining whether regular cannabis use from 13 until 22 years mediated the association between AffI and PD at age 24, path analysis model fit indices indicated good model fit (χ2 = 1.88, *P*=.60, root mean square error of approximation 0, comparative fit index 1.00). Consistent with the adjusted logistic regression, AffI at 12 years old was directly and significantly associated with PD at age 24 (β = 0.028, *P*<.001) (Figure 2A). Further, we observed an indirect effect of regular cannabis use between 13 and 22 years in the association between exposure and PD (bias-corrected estimate = 0.005 [95% CI 0.003–0.007], *P*=.01). The mediating role of regular cannabis use between 13 and 22 years in the association between AffI at age 12 and PEs at age 24 also demonstrated a good model fit (χ2 = 9.7, *P*=.08, root mean square error of ~0.01, comparative fit index 0.99). AffI was directly and significantly associated with PEs at age 24 (β = 0.048, *P*<.001) (Figure 2B). Similarly, we found evidence of an indirect effect of regular cannabis use between 13 and 22 years in the association between exposure and PEs (bias-corrected estimate 0.013 [95% CI 0.009–0.018], *P*=.003).

When occasional users were included as a distinct category, the total indirect effect of AffI on psychotic experiences via cannabis trajectories remained small (NIE ≈ 0.006; ~12% of the association), with the regular-use pathway contributing more than the occasional-use pathway. For psychotic disorder, the estimated indirect effect was also small (~14%, exploratory), noting limited precision due to few cases after complete-case restriction.

## Discussion

This study investigated the longitudinal associations between AffI in early adolescence and psychosis in early adulthood and the potential mediating role of regular cannabis use across adolescence. We provide evidence that AffI is associated with psychotic outcomes at both disorder and symptom level at age 24. We additionally demonstrate that this association may in part be mediated by regular cannabis use throughout adolescence. Our novel results provide insight into the potential mechanisms underlying the longitudinal association between AffI and the interaction of risk factors cumulatively for psychosis from early adolescence to young adulthood.

Our primary finding revealed that AffI at the age of 12 is associated with increased odds of developing either psychotic experiences or psychotic disorder by age 24, compared with individuals showing no evidence of AffI at age 12. This aligns with previous research linking AffI to adverse outcomes across a spectrum of psychiatric disorders, extending our understanding to adolescence and early adulthood.^3^^,^^5^^,^^30^^,^^38^^,^^39^ Notably, our study emphasizes the additional risk that AffI in early adolescence carries for psychosis in early adulthood, highlighting the potential for targeted interventions for AffI during adolescence to mitigate the risk of future psychosis. Given that AffI is transdiagnostic and associated with many psychiatric outcomes, its predictive value is not specific to psychosis. The single-item measure used here likely captures general emotional dysregulation rather than a psychosis-specific risk marker.

Further, our investigation revealed distinct patterns of cannabis use within the cohort from ages 13 to 22 and underlines the significant impact of adolescent cannabis use on development of psychotic outcomes at age 24. Reported prevalences of cannabis use identified during our analysis were consistent with contemporary national figures by age group.^40^^,^^41^ Regular cannabis users had higher odds of experiencing PEs and PD in early adulthood compared with non-users of cannabis during adolescence. These findings corroborate the existing literature implicating cannabis use as a risk factor for various mental health disorders.^20^^,^^42^^,^^43^ Our study underscores the need for targeted interventions addressing cannabis misuse, especially in the prevention of regular use during adolescence and early adulthood, to reduce the substantially elevated risk of individuals developing psychotic outcomes and the potential for addressing AffI therein.

Our findings suggest a significant partial mediation effect of regular cannabis use in the association between AffI in early adolescence and psychosis in early adulthood. Including occasional users as a mediator category did not materially change conclusions: any mediation by adolescent cannabis use appeared partial and was primarily attributable to the regular-use pathway, consistent with the literature linking more frequent use to higher psychosis risk. One interpretation of this finding is that AffI in early adolescence may amplify the risk of psychosis in early adulthood from regular cannabis use throughout adolescence and early adulthood. Given the well-established association between regular cannabis use and psychosis, our study sheds further light on the interplay between these factors in influencing mental health outcomes.

Several potential mechanisms may explain the link between AffI, regular cannabis use, and the subsequent development of psychosis. Regular cannabis use during adolescence has been shown to interfere with brain maturation processes, potentially disrupting functional connectivity in regions such as the prefrontal cortex and hippocampus, areas which are critical for cognitive and emotional regulation.^20^ The presence of AffI could also signal an especially vulnerable subgroup with preexisting atypical brain development patterns, suggesting these individuals may be more susceptible to the neurodevelopmental impacts of regular cannabis use, further heightening the risk of psychotic outcomes. It is also possible that emerging subclinical psychotic symptoms influenced cannabis initiation or escalation, which cannot be ruled out in this design.

Another plausible explanation is that individuals experiencing AffI may turn to regular cannabis use as a form of self-medicating against distressing mood disturbances, anxiety, or other affective challenges. This self-medication, however, may inadvertently amplify the risk of developing psychotic symptoms by increasing cannabis exposure during critical periods of brain development. Collectively, these factors highlight the complex interplay between AffI and regular cannabis use in influencing mental health outcomes and underscore the need for targeted early interventions in this group. These findings support early screening for AffI and early prevention of regular cannabis use as potential intervention targets.

### Strengths and Limitations

Our study represents a novel approach in demonstrating the connection between AffI, psychosis, and regular cannabis use. Our findings highlight the potential role of regular cannabis use as a mediator in the association between AffI and psychosis, suggesting interventions targeting early cannabis use could help mitigate the risk of psychosis, especially in individuals with high AffI. This work has several limitations. First, there is a comparatively short interval between the final cannabis use measurement (age 22) and the timepoint at which psychiatric outcomes were assessed (age 24). A longer interval may have permitted greater distinction between exposure and outcome. This, however, is a trade-off balanced against the greater confidence provided by including more timepoints in the observed trajectories of cannabis use. Second, the outcome measure of psychotic disorder was derived from psychotic-like symptoms scales, as opposed to formal diagnoses of a psychotic disorder made by experienced clinicians against defined criteria from a diagnostic manual, potentially limiting the direct clinical relevance of our findings. The 1.2% prevalence of derived psychotic disorder at age 24 aligns with expected population estimates for early adulthood (0.7–1.5%). However, because the outcome is derived from PLIKSi rather than a structured diagnostic interview, results should not be interpreted as formal diagnoses. Third, though respondents were assured responses regarding potentially sensitive topics such as cannabis use were confidential, it is possible respondents may still have feared repercussions and consequently under-reported cannabis use. Finally, the measurement of AffI relied on a binary classification at a single time point rather than a detailed, specific assessment. A major limitation is the use of a single interviewer-coded CI-BPD item to operationalize AffI. This measure does not capture the multidimensional or fluctuating nature of AffI and its test–retest reliability in adolescence is unknown. The value of using ALPSAC data for this investigation lies in its breadth, permitting longitudinal follow-up across many individuals over multiple years while enabling static and dynamic risk factors to be controlled for. Ultimately, ALPSAC is not a bespoke mental health resource and included measures must balance granularity with utility. Ideally, adolescents would have undergone a comprehensive assessment specifically for AffI. Our method has, however, been used before to explore associations of AffI with psychotic outcomes^5^^,^^30^ and its use in this analysis represents the best available compromise to leverage ALSPAC data for further exploration of potential associations between AffI and later-life psychotic outcomes. Where feasible, future research should employ comprehensive assessments of longitudinal AffI and outcomes of interest.

## Conclusion

This study provides longitudinal evidence for the association of AffI and cannabis use with adult psychosis. Our results highlight the potential importance of early detection and intervention for AffI during adolescence to reduce the prevalence of regular cannabis use and subsequent psychotic sequelae in early adulthood. Further research is needed to explore the reciprocal associations between AffI, regular cannabis use and psychosis, and a detailed mechanistic understanding is required to pave the way for effective prevention and treatment strategies for patients.

## Supplementary Material

Supplementary_Material_accepted_draft_sgag008
