# Supplementary material for: Affective Instability and Regular Cannabis Use During Adolescence and Subsequent Risk for Psychosis in Early Adulthood: A Longitudinal Birth Cohort Study
Source: Schizophr Bull Open. 2026 Mar 18;7(1):sgag008. doi: 10.1093/schizbullopen/sgag008 (PMC13131214; doi:10.1093/schizbullopen/sgag008)
Supplement: Supplementary_Material_accepted_draft_sgag008 [file supplementary_material_accepted_draft_sgag008.docx]

**Supplementary Material**

| **Age** | **13 years** | **14 years** | **15 years** | **17 years** | **20 years** | **22 years** |
| --- | --- | --- | --- | --- | --- | --- |
| Source | Computer in clinic | Postal questionnaire | Computer in clinic | Computer in clinic | Postal questionnaire | Postal questionnaire |
| Do not use | “Have you ever tried cannabis?” = “No” | “Have you ever tried cannabis?” = “No” | “Have you ever tried cannabis?” = “No”  Or, if “yes” to stem, “I have only ever tried cannabis once or twice” or “I used to sometimes use or take cannabis but I never do now” | Have you ever tried cannabis? = “No” | “Have you ever tried cannabis?” = “No” | “Have you ever tried cannabis?” = “No” |
| Occasional | If “yes” to stem, “How many times in the last 6 months?” = “1-3 times” or “>4 times” | If “yes” to stem, “How many times in the last 6 months?” = “1-3 times” or “>4 times” | If “yes” to stem, “I sometimes take cannabis but less often than once a week” | If “yes” to stem,  cannabis use in past 12 months = "Monthly of less" or 2-4 times per month" | If “yes” to stem, “In the last 12 months, how often have you used cannabis?” “Once or twice” or “Less than monthly” or “Monthly (but less than weekly)” “NOT in past 12 months” | If “yes” to stem, “In the last 12 months, how often have you used cannabis?” “Once or twice” or “Less than monthly” or “Monthly (but less than weekly)” “NOT in past 12 months” |
| Frequent | If “yes” to stem, “How many times in the last 6 months?” = “1 x per week” | If “yes” to stem, “How many times in the last 6 months?” = “1 x per week” | If “yes” to stem, “I usually use or take cannabis between one and six times a week” or “I usually use or take cannabis more than six times a week, but I don't use it every day” or “I usually use or take cannabis every day” | If “yes” to stem,  cannabis use in past 12 months = “2-3 times per week” or “4+ times per week” | If “yes” to stem, “In the last 12 months, how often have you used cannabis?”  “Weekly” or  “Daily or almost daily” | If “yes” to stem, “In the last 12 months, how often have you used cannabis?”  “Weekly” or  “Daily or almost daily” |

**Supplementary Table 1.** Recoding of cannabis use questions at each time-point into variables that were used in analyses.

|  | **Non-participating group in the study** | | **Participating group in the study (at 24 years old)** | | | **Non-participating versus participating** | |
| --- | --- | --- | --- | --- | --- | --- | --- |
|  | *Mean* | *SD* | *Mean* | *SD* | | *OR (95% CI)* | *p* |
| Maternal age at delivery | 27.49 | 4.99 | 29.45 | 4.56 | | 1.08 (1.07, 1.09) | <0.001 |
| Gestational age | 38.00 | 6.22 | 39.49 | 1.80 | | 1.09 (1.08, 1.11) | <0.001 |
| Birth weight, kg | 3371.23 | 596.18 | 3410.33 | 532.74 | | 1.25 (1.05, 1.20) | <0.001 |
| Family Adversity Index | 4.74 | 4.47 | 3.61 | 3.84 | | 0.94 (0.93, 0.95) | <0.001 |
|  | **Non-participating group in the study** | | **Participating group in the study** | | |  |  |
|  | *N* | *%* | *N* | | *%* |  |  |
| Sex  Male / Female | 6233 / 4919 | 55.9 / 44.1 | 1458 / 2429 | | 37.5 / 62.5 | 0.47 (0.44, 0.51) | <0.001 |
| Ethnic group  White / Other | 8657 / 248 | 97.2 / 2.8 | 3405 / 78 | | 97.8 / 2.2 | 1.25 (0.97, 1.62) | 0.089 |

**Supplementary Table 2.** Demographic variables of participating and non-participating subjects in the study.

| **Variables** | **Non-users** | **Occasional Users** | **Regular Users** | **F/chi** |
| --- | --- | --- | --- | --- |
| Sex  (n = 5698)(%)  Male/Female | 1365 (46.5%)/  1569 (53.5%) | 1099 (42.7%)/  1494 (57.6%) | 109 (63.7%)/  62 (36.3%) | 34.12*** |
| Ethnic group  (n = 2790)  White/Others | 1371 (98.1%)/  26 (1.9%) | 1272 (97.5%)/  32 (2.5%) | 86/<5 | 1.71 |
| Affective Instability at 12 years age  n= 4789 (Yes/No) | 93 (3.8%)/  2356 (96.2%) | 112 (5.1%)/  2074 (94.9%) | 15 (9.7%)/  139 (90.3%) | 14.25*** |
| Gestational age  at birth in weeks  n = 5432  mean (SD) | 39.42 (1.87) | 39.48 (1.82) | 39.49 (1.92) | 0.77 |
| Family adversity index  n= 5065  mean (SD) | 3.49 (3.71) | 3.72 (3.82) | 5.64 (5.39) | 24.28*** |
| Age of mother at delivery  n=5432  mean (SD) | 28.92 (4.43) | 29.68 (4.57) | 29.40 (4.92) | 18.85*** |
| ***p-value <0.001 | | | | |

**Supplementary Table 3**. Characteristics of study participants at 24 years of age by frequency of cannabis use over preceding 12 years..

| **Composite score of Cannabis Use** | **AIC** | **BIC** | **VLMR-P** | **Entropy** |
| --- | --- | --- | --- | --- |
| 2 classes | 29197.206 | 29239.661 | <0.001 | 0.617 |
| 3 classes | 27974.371 | 28038.053 | <0.001 | 0.728 |
| 4 classes | 27705.856 | 27790.765 | 0.0193 | 0.780 |
| 5 classes | 27300.975 | 27407.111 | 0.2793 | 0.792 |
| 6 classes | 27305.619 | 27432.983 | <0.001 | 0.673 |
| BIC, Bayesian information criterion; VLMR, Vuong-Lo-Mendell-Rubin. | | | | |

**Supplementary Table 4**. BIC, VLMR Likelihood Test p Values, and entropy for Classes 2–6 of cannabis use frequency.
